# Antimicrobial-Resistant Enteric Gram-Negative Bacteria Isolated from a Fatal Diarrhea in a Horse: Genomic Characterization of CTX-M-2-Producing *Escherichia coli*

**DOI:** 10.3390/antibiotics14121185

**Published:** 2025-11-21

**Authors:** Gabriel Augusto Marques Rossi, Fábio Parra Sellera, Carolina Magri Ferraz, Renan Silva de Carvalho, Alvaro de Paula Lage de Oliveira, Camila Angela Marques, Enzo Bernardes Rocha Fávaro, Rafael da Silva Rosa, Leandro Augusto Mariano Silva, Marita Vedovelli Cardozo, Eliana Guedes Stehling, João Pedro Rueda Furlan

**Affiliations:** 1 Department of Veterinary Medicine, University Vila Velha (UVV), Av. Comissário José Dantas de Melo, n.21, Vila Velha 29102-920, ES, Brazil; carolina.ferraz@uvv.br (C.M.F.); renan.carvalho@uvv.br (R.S.d.C.); alvaro.lage@uvv.br (A.d.P.L.d.O.); camila.marques@uvv.br (C.A.M.); enzobernardes@hotmail.com (E.B.R.F.); 2 School of Veterinary Medicine, Metropolitan University of Santos, Santos 11045-002, SP, Brazil; fsellera@usp.br; 3 Department of Internal Medicine, School of Veterinary Medicine and Animal Science, University of São Paulo, São Paulo 05508-220, SP, Brazil; 4 Department of Clinical Analyses, Toxicology and Food Science, School of Pharmaceutical Sciences of Ribeirão Preto, University of São Paulo, Ribeirão Preto 19040-903, SP, Brazil; rafaelsilvarosa@usp.br (R.d.S.R.); elianags@usp.br (E.G.S.); 5 Department of Pathology, Reproduction and One Health, Faculty of Agricultural and Veterinary Sciences, São Paulo State University (UNESP), Jaboticabal 14884-900, SP, Brazil; leandro.am.silva@unesp.br (L.A.M.S.); marita.vedovelli@unesp.br (M.V.C.); 6 Department of Pharmaceutical Sciences, Health Sciences Center, Federal University of Paraíba, João Pessoa 58051-900, PB, Brazil; furlanjpr@gmail.com

**Keywords:** *Enterobacterales*, WHO critical priority pathogen, ESBL, equine medicine

## Abstract

**Background:** Infections caused by antimicrobial-resistant bacteria are difficult to treat and increase the risk of death in animals. This report describes a fatal case of diarrhea in a horse that, despite intensive treatment including surgery and broad-spectrum antimicrobials (ceftiofur and amikacin), experienced a worsening of its condition and subsequent death. **Methods:** A fecal swab sample was subjected to microbiological culture for the identification of bacteria and assessment of their phenotypical antimicrobial susceptibility profiles using the disk-diffusion and broth microdilution methods. The double-disk synergy test, polymerase chain reactions for the detection of genes encoding extended-spectrum β-lactamases, and whole-genome sequence-based analysis were also performed. **Results:** Strains of *Salmonella enterica* and *Escherichia coli* were isolated, with the *E. coli* strain DSL-HVUVV-2025 presenting resistance to a third-generation cephalosporin. Accordingly, the *bla*_CTX-M-2_ gene was identified in the DSL-HVUVV-2025 strain, which was submitted to whole-genome sequencing. Genomic analysis showed several antimicrobial resistance determinants, as well as virulence genes, including those associated with the enteroaggregative pathotype. The *bla*_CTX-M-2_ gene was surrounded by an IS*CR1* element and embedded in a complex class 1 integron that is part of the Tn*7337* transposon. Strain DSL-HVUVV-2025 belonged to a novel sequence type. **Conclusions:** This case highlights the importance of monitoring antimicrobial resistance and performing genomic characterization of bacteria involved in equine diarrhea to guide effective clinical management in veterinary hospitals. It also reinforces the role of horses as potential carriers of WHO critical priority pathogens and the need for responsible antimicrobial use.

## 1. Introduction

Enterocolitis is one of the most frequent and severe gastrointestinal disorders in horses, often leading to fatal outcomes. Determining the causative agent is particularly difficult because several pathogens can produce overlapping clinical signs and similar pathological lesions. Moreover, many of these microorganisms may also be present in the intestines of clinically healthy horses, further complicating the diagnosis. The most common infectious agents associated with equine enterocolitis include *Salmonella* spp., *Clostridium* spp., *Paeniclostridium sordellii*, *Rhodococcus equi*, *Neorickettsia risticii*, *Lawsonia intracellularis*, equine rotavirus, and equine coronavirus. In general, diagnosis relies on a combination of clinical, gross, and histopathological findings, and is confirmed by identifying the pathogen or its toxins in fecal or intestinal samples [1]. The treatment of acute diarrhea in horses remains largely empirical and primarily supportive until specific diagnostic test results are available. Supportive therapy focuses on restoring hydration, correcting electrolyte imbalances, and controlling the systemic inflammatory response. Antimicrobials are frequently administered to manage infectious processes; however, their use can disrupt the intestinal microbiota, leading to dysbiosis. Therefore, antimicrobial therapy should be guided by culture and susceptibility testing of the causative agents [2], given the widespread occurrence of antimicrobial-resistant strains.

The global crisis of antimicrobial resistance (AMR) poses a serious and escalating threat to human and animal health, as well as their shared environment [3]. Among the various antimicrobial-resistant pathogens, Gram-negative bacteria stand out due to their remarkable ability to acquire and disseminate multiple antimicrobial resistance genes (ARGs) [4]. These bacteria often harbor mobile genetic elements, such as plasmids and transposons, that facilitate the horizontal transfer of AMR determinants across species and genera, further complicating treatment options and contributing to the global burden of difficult-to-treat infections [5].

*Salmonella enterica* and *Escherichia coli* are clinically important Gram-negative bacteria and important reservoirs of acquired ARGs, with specific lineages often associated with life-threatening enteric and systemic infections [6,7]. *E. coli*, a commensal microorganism in the gastrointestinal tract of many species, has emerged as a major reservoir and disseminator of extended-spectrum β-lactamases (ESBL), enzymes that hydrolyze broad-spectrum cephalosporins [7]. *S. enterica* is a globally significant zoonotic pathogen, often associated with fluoroquinolone resistance and foodborne outbreaks [8].

Despite increasing reports of Gram-negative enteric infections in horses [1], equine medicine remains relatively underrepresented in AMR research. Although awareness of antimicrobial-resistant bacterial infections in equine patients is growing [9,10,11], their true clinical relevance and genetic diversity are likely still underestimated, largely due to the scarcity of comprehensive epidemiological and genomic investigations. These knowledge gaps hinder the understanding of resistance dynamics and the implementation of evidence-based antimicrobial stewardship in equine medicine. In this context, the present study reports a fatal case of diarrhea in a horse from which two antimicrobial-resistant bacteria were isolated, including a third-generation cephalosporin-resistant *E. coli* strain. This study aimed to describe the clinical course and genomic characteristics of a CTX-M-2-producing *E. coli*, a critical priority pathogen according to the World Health Organization (WHO), highlighting its antimicrobial resistance and virulence profiles through whole-genome sequencing (WGS) and discussing their implications for equine health and antimicrobial stewardship.

## 2. Clinical Case Description

### 2.1. History, Clinical Evaluation, and Conservative Treatment

A 6-year-old, 430 kg male Mangalarga Marchador breed from Domingos Martins, Espírito Santo, Brazil, was referred to a large animal hospital with a 3-day history of abdominal discomfort, as reported by the animal’s owner. Initial treatment by a local veterinarian included nasogastric intubation, intravenous fluid therapy, and flunixin meglumine, but there was no clinical improvement. The horse was kept in a stall with paddock access and fed chopped grass, silage, and commercial concentrate. Vaccination and deworming status were unknown.

On the first day the animal was admitted to the veterinary hospital, the horse showed mild tachycardia (44 bpm; normal ≤ 40 bpm) [12], an increased respiratory rate (32 breaths/min), and clinical signs of mild to moderate dehydration with reduced peripheral perfusion, including pink oral mucosa, a capillary refill time of three seconds, and a skin tent of two seconds. Abdominal examination revealed hypomotility in the upper left quadrant and altered intestinal positioning, with the spleen displaced medially and the left kidney not palpable or visualized on ultrasound. No gastric reflux was detected, and hematological and biochemical parameters were within reference ranges. A conservative approach was initiated with intravenous fluids, Sedacol^®^ (Calbos^®^ Saúde Animal, São José dos Pinhais, Brazil) (100 mL q12h), warm water via nasogastric tube (2 L/h), and controlled walking. After 24 h of its admission, the horse developed bilateral abdominal distension and moderate colic unresponsive to analgesia, necessitating exploratory laparotomy.

### 2.2. Surgical Intervention and Postoperative Management

Under general inhalation anesthesia and dorsal recumbency, a ventral midline incision was performed. An atypical nephrosplenic entrapment of the large colon was identified, with medial displacement and splenomegaly, and rounded splenic margins consistent with compression. An enterotomy was performed at the pelvic flexure for decompression, followed by enterorrhaphy and abdominal lavage with povidone-iodine and sterile saline. The colon was repositioned, and the abdominal wall was closed in layers using polyester (linea alba), Monocryl^®^ (Ethicon^®^, São Paulo, Brazil) (subcutaneous tissue), and nylon (skin).

Postoperative care included ceftiofur (5 mg/kg IV SID × 5 days), gentamicin (6.6 mg/kg IV SID × 5 days), metronidazole (30 mg/kg IV SID × 4 days), flunixin meglumine (1.1 mg/kg IV SID × 5 days), 10% DMSO (IV SID × 5 days), omeprazole (4 mg/kg PO SID × 5 days), and heparin (40 IU/kg SC TID × 3 days). Supportive measures included probiotics, activated charcoal, oral electrolytes, thiamine, and cyanocobalamin. A continuous lidocaine infusion was administered for the first three postoperative days (1.3 mg/kg bolus over 15 min followed by 3 mg/kg/h for 9 h). After 72 h postoperatively, the horse presented with fever (39 °C), apathy, and recurrent abdominal pain, accompanied by diarrhea that initially appeared soft and pasty but rapidly progressed to profuse, watery, and fetid stools.

### 2.3. Bacterial Obtaining and Clinical Case Evolution

A fecal swab was collected and seeded on Salmonella-Shigella agar (Kasvi, Imperia, Italy), MacConkey Agar (Kasvi, Madrid, Spain), and Sheep Blood Agar Base (HiMedia, Thane, India). The plates were incubated at 35 ± 2 °C in an aerobic atmosphere for 18–24 h. Macroscopically different colonies were selected, and the bacterial identification was carried out using Gram staining and the Bactray system (Laborclin^®^, Pinhais, Paraná, Brazil) [13]. Accordingly, three strains belonging to different species were identified as follows: *Proteus mirabilis*, *E. coli*, and *S. enterica*. Although *P. mirabilis* was isolated, it was not further investigated, as this species is commonly part of the intestinal microbiota and is not typically associated with diarrheagenic syndromes [14].

Initial antimicrobial susceptibility testing (AST) was performed using the disk diffusion method, as described by BrCAST and CLSI, with *E. coli* ATCC^®^ 25922 serving as the quality control strain [15,16]. Briefly, bacterial strains were cultured on nutrient agar plates (Kasvi^®^, Pinhais, Curitiba, Brazil) and incubated at 35 ± 2 °C in an aerobic atmosphere for 18–24 h. Bacterial suspensions equivalent to 0.5 McFarland standard were then prepared using the Densimat Densitometer (bioMérieux, Craponne, France). The suspensions were subsequently seeded onto Mueller–Hinton agar plates (Kasvi, Spain) with a sterile swab. Antimicrobial disks, including trimethoprim/sulfamethoxazole (1.25/23.75 µg), amikacin (30 µg), gentamicin (10 µg), meropenem (10 µg), and ceftiofur (30 µg) (Cecon, Vitória, Brazil), were placed on the surface. Finally, the plates were incubated at 35 ± 2 °C in an aerobic atmosphere for 16–18 h, and the inhibition zone diameters were measured and interpreted.

In vitro results showed that *E. coli* and *S. enterica* strains were resistant to trimethoprim/sulfamethoxazole and gentamicin but remained susceptible to amikacin and meropenem. Notably, the *E. coli* strain also exhibited resistance to ceftiofur, whereas the *S. enterica* strain remained susceptible to this antimicrobial. Based on initial AST results, gentamicin was replaced with amikacin (20 mg/kg SID). Follow-up hematologic analyses (URIT 300 Plus^®^, MHLab, São Paulo, Brazil) revealed no abnormalities; however, serum biochemistry (Humastar 80^®^, InVitro, São Paulo, Brazil) showed elevated AST (459 U/L), GGT (21.9 U/L), total bilirubin (9.19 mg/dL), and indirect bilirubin (9.0 mg/dL), indicating a cholangiohepatitis secondary to intestinal disease or to drug administration during the management of the clinical case. Hemoparasite testing by ear tip smear was negative. Ultrasonography demonstrated significant fluid accumulation in the small intestine and large colon. Diarrhea, fever, and depression persisted, requiring ongoing fluid therapy and intensive care. On the fifth postoperative day (six days after the animal’s admission), the horse developed marked abdominal distension and severe, unresponsive colic. Despite aggressive management, the animal deteriorated and died.

### 2.4. In-Depth Investigation of Ceftiofur-Resistant E. coli Strain

The WHO has classified species belonging to the order *Enterobacterales* that are resistant to third-generation cephalosporins as critical priority pathogens [17]. Accordingly, the *E. coli* strain, denominated DSL-HVUVV-2025, was submitted to an additional AST using broth microdilution methods [15,16]. Strain DSL-HVUVV-2025 was also resistant to ceftriaxone (MIC > 64 mg/L), ceftazidime (MIC 64 mg/L), cefepime (MIC > 64 mg/L), aztreonam (MIC 32 mg/L), nalidixic acid (MIC 64 mg/L), ciprofloxacin (MIC 32 mg/L), and tetracycline (MIC 64 mg/L). The double-disk synergy test was performed [18] and showed the phenotypic production of ESBL. Genomic DNA was extracted by PureLink™ Genomic DNA Mini Kit (Thermo Fisher Scientific, Waltham, MA, USA) according to the manufacturer’s recommendations, and molecular detection of *bla*_CTX-M-Gp2_ gene was performed by polymerase chain reactions as previously described [19].

ESBL-positive *E. coli* strain DSL-HVUVV-2025 was submitted to WGS using Oxford Nanopore Technologies. The draft genome was de novo assembled, quality checked, and annotated using Flye v.2.9.1 (https://github.com/mikolmogorov/Flye, accessed on 20 June 2025). CheckM v.1.2.4 (https://github.com/Ecogenomics/CheckM, accessed on 20 June 2025) and NCBI Prokaryotic Genome Annotation Pipeline (https://github.com/ncbi/pgap, accessed on 20 June 2025), respectively. Bioinformatic analyses were conducted using tools available at the Center for Genomic Epidemiology (http://www.genomicepidemiology.org/, accessed on 20 June 2025) and ClermonTyping (http://clermontyping.iame-research.center/, accessed on 20 June 2025). All bioinformatic platforms and pipelines were used with default parameters.

Strain DSL-HVUVV-2025 (GenBank accession number: JBPVNZ000000000) belonged to phylogroup E, serotype O188:H34, and carried the *fimH*61 allele. A new sequence type (ST) with the closest profile (nearest match: 2 *loci*) to ST954, ST1266, ST4953, ST5930, ST11994, and ST17723, was identified. This ST presented known alleles of *adk* (827), *gyrB* (1629), *mdh* (24), and *purA* (85) and new alleles of *fumC*, *icd*, and *recA* (Table 1). This new ST could not be assigned because EnteroBase [20] only validates short-read data, whereas our sequences were obtained using the long-read technology.

This strain harbored acquired ARGs to β-lactams (*bla*_CTX-M-2_), aminoglycosides [*aadA1*, *aac(3)*-VIa], sulfonamides (*sul1*, *sul2*), tetracyclines (*tetA*, *tetB*), amphenicols (*cmlA1*), and fosfomycin (*fosA3*). Mutations in fluoroquinolone resistance determinants, including S83L and D87N in GyrA and S80I and E84G in ParC, were identified. These genotypic results corroborate the multidrug resistance phenotype observed. Furthermore, resistance genes to benzylkonium chloride (*qacE*) and hydrogen peroxide (*sitABCD*) were also found.

Multiple virulence-associated genes were detected, including adhesion and colonization determinants (*air*, *F17A*, *F17C*, *F17D*, *F17G*, *fimH*, *lpfA*, *yehABCD*, *csgA*, *hra*, *fdeC*), invasion-related elements (*tia*), toxins and modulators (*hlyE*, *hha*, *tsh*, *yghJ*), iron acquisition systems (*chuA*, *fyuA*, *iroN*, *irp2*, *sitA*, *iucC*, *iutA*), and persistence and stress adaptation factors (*gad*, *traT*, *terC*, *shiA*). Among these, *air* (encoding the enteroaggregative immunoglobulin repeat protein), *tia* (invasion determinant), and *yghJ* (a mucin-degrading metalloprotease linked to enhanced intestinal inflammation and fluid loss) stand out as the most relevant virulence factors detected, particularly when acting synergistically with adhesion-related genes (*F17* cluster, *fimH*, *lpfA*).

Plasmid replicons IncFIB(AP001918), IncFIC(FII), IncI1-Iα, and Col440I were found. The *bla*_CTX-M-2_ gene was surrounded by an IS*CR1* element and embedded in a complex class 1 integron that is part of the Tn*7337* transposon (GenBank accession number: MZ396394). By using BLASTn analysis (https://blast.ncbi.nlm.nih.gov/Blast.cgi, accessed on 4 July 2025), this sequence presented >99.8% nucleotide identity to other IncI1-Iα plasmid sequences, predicting its plasmid location.

## 3. Discussion

In this report, we describe a fatal case of diarrhea in a horse, in which two distinct antimicrobial-resistant Gram-negative species were isolated. In the clinical case, it was not possible to definitively identify the etiologic agent responsible for diarrhea. Testing for viruses, parasites, or bacterial toxins was not conducted, and the detection of *S. enterica* and *E. coli* alone, without supporting histopathological analysis, does not allow for a conclusive diagnosis, given the complexity of potential causative agents in equine enterocolitis [1]. Other etiological agents that can cause enterocolitis in horses and could have been responsible for the infection include bacteria such as *Clostridium* spp., *Paeniclostridium sordellii*, *Rhodococcus equi*, *Neorickettsia risticii*, and *Lawsonia intracellularis*, which were not isolated in the culture media, as well as viruses such as equine rotavirus and equine coronavirus [1]. Nevertheless, *E. coli* and *S. enterica* are recognized as possible contributors, and the lack of response to empirical broad-spectrum therapy, initially involving third-generation cephalosporins and aminoglycosides, suggests that these antimicrobial-resistant bacteria may have played a role in the therapeutic failure and progression of the disease. In this regard, despite prompt surgical intervention and intensive postoperative care, the horse rapidly deteriorated, with persistent diarrhea, fever, and signs of endotoxemia leading to the animal’s death.

The identification of *E. coli* and *S. enterica* resistant to multiple critically important antimicrobials highlights the emergence of multidrug-resistant (MDR) clones in equine populations. Infections caused by antimicrobial-resistant bacteria are associated with increased treatment failures, higher morbidity and mortality, longer hospital stays, and elevated overall healthcare costs [21]. Ceftiofur is widely used in equine medicine for its broad antibacterial activity and favorable safety profile. It is often employed to prevent and treat common infections; however, its use should be restricted, as it has been linked to antimicrobial-associated colitis in horses. Clinicians should reserve ceftiofur for cases where first-line antimicrobials are unsuitable. As a medically important drug with reported bacterial resistance in horses, its use requires careful antimicrobial stewardship [22]. However, since the *E. coli* strain demonstrated resistance to this antimicrobial, antimicrobial therapy was carried out using amikacin, a widely used drug in horses to which the strain showed susceptibility [23].

*E. coli* is a bacterium with remarkable genomic plasticity, allowing it to adapt to diverse ecological niches and cross host barriers through interspecies transmission [24]. In addition to its role as a commensal, diarrheagenic *E. coli* strains have been linked to enteric diseases in animals, including horses [25,26]. The detection of *E. coli* is increased in horses with intestinal diseases, such as large intestinal colic [27]. Virulence determinants were found in the strain DSL-HVUVV-2025, including those typically found in enteroaggregative *E. coli* (*air*) and enterotoxigenic *E. coli* (*yghJ*) pathotypes, and may have contributed to the diarrhea [28,29]. Additionally, the F17 virulence factor may play a role in causing diarrhea in horses and was detected in our strain [30]. Importantly, the convergence of virulent factors and multidrug resistance has been increasingly documented in *E. coli* strains, constituting an additional threat to public health [31,32].

*S. enterica* has been primarily associated with nosocomial outbreaks [33] and is a well-recognized cause of enterocolitis in horses. A conclusive diagnosis, beyond the observation of clinical signs, requires the laboratory detection of *Salmonella* spp. in fecal samples or intestinal contents [1]. In horses, the most frequently reported serotypes are the zoonotic *S.* Typhimurium and *S.* Enteritidis. These serotypes are widely recognized for causing gastroenteritis in multiple hosts and often described as resistant to gentamicin, amikacin, ampicillin, ceftazidime, ceftiofur, chloramphenicol, and trimethoprim/sulfamethoxazole [34,35]. In this case, although the strain was not fully characterized through serotyping or genomic analysis, it exhibited resistance to clinically important antimicrobials. Its coexistence with a virulent and ESBL-producing *E. coli* strain may have contributed to disease severity, potentially enhancing intestinal inflammation and compromising gut integrity.

Notably, *E. coli* strain DSL-HVUVV-2025 was identified as carrying the *bla*_CTX-M-2_ gene, which is one of the most prevalent and clinically significant ESBL variants in Brazil [36]. Worryingly, the occurrence of ESBL-producing strains has been increasingly reported in horses. *E. coli* strains harboring ESBL genes were identified in fecal samples [37], including those collected upon arrival at clinics [38] and in equine specialty hospitals and their shared environments [39,40,41]. In Brazil, two cases of ESBL-producing *E. coli* infecting horses were described. The first involved a CTX-M-15-positive *E. coli* ST2179 isolated from an extraintestinal infection. In this case, the animal exhibited watery diarrhea, hyperthermia (41 °C), abdominal distension, colic, weakness, and poor body condition, and died two days after admission. Liver samples yielded a ceftiofur-resistant *E. coli* strain upon microbiological examination [42]. The second case also involved an extraintestinal infection, caused by a CTX-M-8-producing *E. coli* ST711 recovered from lung tissue collected during the necropsy [43]. However, the detection of CTX-M-2-carrying *E. coli* has been found in horses from Japan [44] and Belgium [45], with the *bla*_CTX-M-2_ gene located on conjugative plasmids.

Remarkably, during a bacterial outbreak at an equine veterinary teaching hospital, factors such as mare-foal pairs, prolonged hospitalization, and the use of nasogastric tubes (as in the present case) were associated with the presence of ESBL-producing strains [46]. Moreover, the detection of ESBL-positive *Enterobacterales* in stomach drench pumps used with nasogastric tubes underscores the importance of adequate cleaning and disinfection protocols [46]. Overall, the prevalence of these strains seems to be higher in hospitalized horses compared to those on farms, and longer hospital stays may further increase this risk. It is worth noting that certain host-related factors, such as breed, male sex (stallions), and a prior history of antimicrobial therapy, have also been associated with higher detection rates of ESBL-producing *Enterobacterales* in horses [47].

From an epidemiological standpoint, this case underscores the need to strengthen surveillance systems for MDR pathogens in equine medicine, given their potential impact on clinical outcomes. Routine implementation of microbiological culture, AST, and even genomic analyses should be considered essential components of equine clinical practice. The identification of multiple resistance and virulence genes in Gram-negative pathogens isolated from horses further underscores concern about their zoonotic potential and their role in the broader dissemination of AMR within a One Health context. Horses may act as reservoirs and disseminators of antimicrobial-resistant bacteria, which have significant implications for human, animal, and environmental health. Their close interaction with humans, as working animals, companions, and participants in therapeutic practices, facilitates the transmission of zoonotic pathogens. Environmental contamination through fecal shedding, respiratory secretions, and direct contact further extends the potential exposure to multiple hosts. These patterns highlight the need for One Health–oriented surveillance and preventive strategies, including antimicrobial stewardship, biosecurity, and handler education, to mitigate AMR dissemination across species and ecosystems [21].

Thus, based on the obtained and discussed results, the detection of a new ST of *E. coli* producing CTX-M-2, together with an antimicrobial-resistant *S. enterica*, in a clinical case of acute diarrhea that rapidly led to the animal’s death, is of concern. The *E. coli* strain carried several virulence genes, including those associated with the enteroaggregative pathotype, as well as multiple AMR determinants, indicating the potential impact of its transmission to other sources. Moreover, its detection in a hospital environment represents a possible health risk to other animals if transmission occurs through improperly disinfected facilities or equipment [46].

This study has limitations that should be considered when interpreting the results: (i) it was not possible to establish a direct causal relationship between the two identified antimicrobial-resistant bacteria in feces and the clinical outcomes; (ii) it was not possible to determine whether the presence of the *E. coli* strain contributed to the worsening of the diarrhea or if it merely represented opportunistic colonization of the compromised postoperative patient, despite carrying important virulence genes; (iii) *S. enterica* was not serotyped and genomic characterization was performed exclusively on the third-generation cephalosporin-resistant *E. coli* strain, as this bacterium is classified as a critical priority pathogen; (iv) necropsy, histopathological examinations, and tests for toxins, parasites, or viruses were not performed, which could have helped confirm the etiology of the fatal case; and (v) metagenomic analyses could have contributed to a better understanding of the animal’s intestinal microbiome and to establishing the relationship between the identified bacteria and the clinical presentation of acute diarrhea.

## 4. Conclusions

This case highlights the importance of assessing the antimicrobial susceptibility profiles and in-depth characterization of etiological agents involved in acute diarrhea in horses to support more effective clinical management, as well as the relevance of equines as carriers of WHO critical priority pathogens. It also highlights the need for targeted interventions in this species to prevent their dissemination to other hosts. Furthermore, this report emphasizes the necessity of routine antimicrobial resistance screening in equine clinical cases and the judicious use of antimicrobials to prevent therapeutic failures and reduce the spread of antimicrobial-resistant strains. The integration of genomic surveillance strategies in equine hospital settings could further enhance early detection and monitoring of emerging multidrug-resistant pathogens, thereby contributing to improved infection control and responsible antimicrobial stewardship.

## Figures and Tables

**Table 1:** MLST profile of *E. coli* strain DSL-HVUVV-2025 and nearest sequence types.

ST ^1^	Achtman Seven-Gene MLST Scheme ^2^						
	*adk*	*fumC*	*gyrB*	*icd*	*mdh*	*purA*	*recA*
954	19	184	53	140	24	85	42
1266	168	184	53	140	24	85	42
4953	168	209	53	140	24	85	42
5930	168	184	5	140	24	85	42
11994	168	184	1145	140	24	85	42
17723	168	184	53	2231	24	85	42
ND	827	398 ^(^*^)^	1629	1209 ^(^*^)^	24	85	42 ^(^*^)^

^1^ ST, sequence type; ND, not determined. ^2 (^*^)^, alleles with less than 100% identity.

## Data Availability

The data presented in this study are openly available in GenBank (GenBank accession number: JBPVNZ000000000).
